# Growth kinetics and fitness of fluoroquinolone resistant and susceptible *Campylobacter jejuni* strains of cattle origin

**DOI:** 10.3389/fvets.2023.1117975

**Published:** 2023-04-18

**Authors:** Debora Brito Goulart, Qijing Zhang, Orhan Sahin

**Affiliations:** ^1^Department of Veterinary Microbiology and Preventive Medicine, Iowa State University, Ames, IA, United States; ^2^Department of Veterinary Diagnostic and Production Animal Medicine, Iowa State University, Ames, IA, United States

**Keywords:** antimicrobial resistance, fluoroquinolone, *Campylobacter*, cattle, mutant selection window, growth rate, bacterial fitness

## Abstract

Human enterocolitis is frequently caused by the Gram-negative microaerobic bacterium *Campylobacter jejuni*. Macrolides (e.g., erythromycin) and fluoroquinolones (FQs) (e.g., ciprofloxacin) are the preferred antibiotics for the treatment of human campylobacteriosis. Rapid emergence of FQ-resistant (FQ-R) *Campylobacter* during treatment with FQ antimicrobials is well known to occur in poultry. Cattle is also an important reservoir of *Campylobacter* for humans, and FQ-R *Campylobacter* from cattle has become highly prevalent in recent years. Even though the selection pressure may have contributed to the expansion of FQ-R *Campylobacter*, the actual impact of this factor appears to be rather low. In this study, we examined the hypothesis that the fitness of FQ-R *Campylobacter* may have also played a role in the rise seen in FQ-R *Campylobacter* isolates by employing a series of *in vitro* experiments in MH broth and bovine fecal extract. First, it was shown that FQ-R and FQ-susceptible (FQ-S) *C. jejuni* strains of cattle origin had comparable growth rates when individually cultured in both MH broth and the fecal extract with no antibiotic present. Interestingly, FQ-R strains had small but statistically significant increases over FQ-S strains in growth in competition experiments performed in mixed cultures with no antibiotic present. Lastly, it was observed that FQ-S *C. jejuni* strains developed resistance to ciprofloxacin more readily at high initial bacterial cell density (10^7^ CFU/mL) and when exposed to low levels of the antibiotic (2–4 μg/mL) compared with that at a low level of initial bacterial cell density (10^5^ CFU/mL) and exposure to a high level of ciprofloxacin (20 μg/mL) in both MH broth and the fecal extract. Altogether, these findings indicate that even though FQ-R *C. jejuni* of cattle origin may have a slightly higher fitness advantage over the FQ-S population, the emergence of FQ-R mutants from susceptible strains is primarily dictated by the bacterial cell density and the antibiotic concentration exposed under *in vitro* condition. These observation may also provide plausible explanations for the high prevalence of FQ-R *C. jejuni* in cattle production due to its overall fit nature in the absence of antibiotic selection pressure and for the paucity of development of FQ-R *C. jejuni* in the cattle intestine in response to FQ-treatment, as observed in our recent studies.

## 1. Introduction

*Campylobacter* is one of the most prevalent causes of bacterial foodborne gastroenteritis worldwide (1, 2). In the United States*, Campylobacter* causes an estimated 1.3 million illnesses and costs ~$1.7 billion yearly for medical treatment and lost productivity (3, 4). Human *Campylobacter* infections are primarily caused by the consumption of contaminated poultry meat (5, 6). In addition to chickens, *Campylobacter* is prevalent in both beef and dairy cattle (7–9). Humans can acquire *Campylobacter* from cattle through direct contact, ingestion of unpasteurized milk, and water contamination (10–15). Although most individuals infected with *Campylobacter* may not require antibiotic treatment, severe and systemic infections necessitate antimicrobial therapy, including macrolides (e.g., erythromycin) and fluoroquinolones (FQs) (e.g., ciprofloxacin) (16–19). Unfortunately, both classes of antibiotics are becoming less effective in treating campylobacteriosis due to increasing rates of resistance to these drugs in *Campylobacter* (20–22). The fact that *Campylobacter* is a zoonotic pathogen exposed to FQs used in both animal production (e.g., beef cattle and non-lactating dairy cattle) and human medicine may contribute to the development of FQ-resistant (FQ-R) *Campylobacter*. In counties like the United States, Australia, and Canada, FQ antibiotics such as enrofloxacin and danofloxacin have indications for subcutaneous use in both sick (therapeutic treatment) and healthy cattle (metaphylaxis) at high risk of bovine respiratory disease (BRD) development (23–29).

Fluoroquinolone-resistant mutant can spontaneously develop in *Campylobacter* (30, 31), and the use of FQ antibiotics selects and enriches these mutants (32). In *Campylobacter*, FQ resistance is mostly caused by point mutations in the quinolone resistance-determining regions (QRDR) of DNA gyrase (*gyrA*) (33, 34), most commonly with the Thr-86-Ile amino acid substitution (C257T mutation), in conjunction with the function multidrug efflux pump CmeABC (34–37). Interestingly, FQ resistance caused by *gyrA* mutations can be maintained in *Campylobacter* without antibiotic selection pressure, suggesting that FQ-R mutants do not carry a fitness burden (38, 39). For example, a previous study conducted by our group revealed a significant fitness advantage of FQ-R over FQ-susceptible (FQ-S) *Campylobacter jejuni* without antibiotic selection pressure when co-inoculated into chickens (40). Interestingly, the fitness change in FQ-R *C. jejuni* could not be attributed to compensatory mutations because no mutations other than the resistance-conferring C257T mutation were found in the *gyrA* and *gyrB* genes of the resistant strains (40).

Because FQ-R *Campylobacter* may still maintain fitness in the absence of antibiotic selection pressure, the reduced or discontinued antimicrobial use in food-producing animals may not necessarily result in an immediate decline in the frequency of FQ-R *Campylobacter*. For example, FQ-R *Campylobacter* was found in 40% of chicken products in two United States companies that had not used FQs for at least 1 year (41). Likewise, FQ-R *Campylobacter* remained for many rotations on Danish broiler farms that had stopped using FQ antibiotics for 4 years (42). In a recent study conducted by our group, it was found that the vast majority of dairy calves (26/30; 87%) were colonized by FQ-R *C. jejuni* even though they had no known previous exposure to FQ antibiotics (32). Similar findings were noted in a study with beef calves in which more than 60% of the *Campylobacter* isolates were resistant to at least one FQ antibiotic (e.g., nalidixic acid or ciprofloxacin) before treatment (43). A study conducted at commercial beef cattle confined feeding operations in Alberta, Canada found a relatively low level of resistance to FQs (^~^5–7%, ciprofloxacin and nalidixic acid) in *C. jejuni* isolates upon feedlot arrival, but the resistance rate significantly increased (to ^~^10–15%) after 60 days of maintenance period at some operations that did not use any FQ antibiotics (44). Interestingly, the same study showed a correlation between FQ resistance and genotype as certain subtypes of *C. jejuni* had higher rates of resistant isolates (44). Intriguingly, a longitudinal research on the incidence of antimicrobial-resistant *Campylobacter* in swine raised without antibiotics discovered a ciprofloxacin resistance rate of 17.1% in *Campylobacter coli* (45). These studies suggest that the fitness of FQ-R *Campylobacter* may contribute to the persistence of FQ resistance in the farm environment of various food-producing animals regardless of antimicrobial usage.

Very recently, we conducted a study with commercial dairy calves to evaluate the effect of subcutaneous (s.c.) administration of a single dose danofloxacin on the development of FQ resistance in *C. jejuni* in both healthy and BRD-induced calves (32). Data from that study showed that most of the calves were naturally colonized by a mixture of FQ-R and FQ-S *C. jejuni* strains (^~^50% of each population) even though these animals were known not to be exposed to FQs previously per the farm records, suggesting that FQ-R strains may have a fitness advantage over FQ-S strains that allowed them to thrive in the gastrointestinal tract of cattle in the absence antibiotic selection pressure. To test this hypothesis, here we performed a series of *in vitro* experiments using both Mueller-Hinton (MH) broth and bovine fecal extract (in an attempt to mimic cattle intestinal tract), including the growth kinetics and competition as well as resistance development, using the FQ-R and FQ-S *C. jejuni* strains collected from the same study (32). It should be noted that natural carriage of *Campylobacter* in the intestine of healthy cattle is common and the organism is usually not associated with any overt disease in cattle (32).

## 2. Methods

### 2.1. Bovine fecal extract

*Campylobacter*-free rectal feces collected freshly and saved at −80°C during our previous investigation (46) was used as a bovine fecal extract in the current study. To confirm the *Campylobacter*-free status, fecal samples were plated on Mueller-Hinton (MH) agar (Difco, BD, Sparks, MD) plates containing *Campylobacter* growth supplement (SR084E; Oxoid, Basingstoke, England) and Preston *Campylobacter* selective supplement (SR117E; Oxoid). Plates were incubated at 42°C for 48 h under microaerobic conditions (10% CO_2_, 5% O_2_, 85% N_2_). Enrichment culture was also performed as described elsewhere (32) to ensure the free *Campylobacter* status of the fecal samples, as this method is more sensitive than direct culture when the number of *Campylobacter* in cattle feces is low (47). Once the free status was confirmed by enrichment, the fecal extract was prepared using the *Campylobacter*-free bovine feces resuspended in MH broth (1:1 in equal volume), and the resuspension was sterilized by a step-wise filtering process (0.80 μm and 0.20 μm pore sized filters; Corning^®^ syringe filters, Millipore Sigma, United States) as described in one of our previous investigations (48). To check for sterility, the filtered feces were plated on MH agar and blood agar plates (5% sheep blood agar) and incubated at 37°C under aerobic and microaerobic conditions for 72 h. Once sterility was confirmed (no growth of any bacterial colony), the filtered bovine fecal extract was stored in 50 mL sterile centrifuge tubes (10 mL per tube) at −80°C until further use.

### 2.2. Bacterial strains and culture conditions

The FQ-S and FQ-R *C. jejuni* strains used in this study are listed in Table 1. The majority of *C. jejuni* strains (*n* = 4; origin: Iowa) were isolated from the feces of healhty calves in our very recent study on FQ-resistance development in experimental cattle (32). These four strains were selected because they belonged to the most common MLST sequence types (ST) colonizing the calves and had different FQ susceptibility phenotypes (32). One (ST-93) of strains was originally isolated from the feces of healhy feedlot cattle (Missouri) in our previous study (7) and was one of the inoculum strains used to inoculate the experimental calves in our recent study (32). This strain (ST-93) was re-isolated from the experimentally inoculated calves in that study (32), and was selected to be included (the re-isolated strain) for use in the current study. The strain NCTC 11168 (49) was originally isolated from a diarrheaic human stool and is a commonly used reference strain by many investigators around the world. All the cattle strains were previously identified to the species level by MALDI-TOF mass spectrometry following the manufacturer’s (Bruker Daltonik, Billerica, MA, United States) instructions and standard operating procedures at the Veterinary Diagnostic Laboratory at Iowa State University (32). Minimum inhibitory concentrations (MICs) of ciprofloxacin for all of the strains were determined using commercial Sensititre CAMPY2 plates (Thermo Fisher Scientific) in our previous study (32); no further standard MIC testing was performed in the current study. Instead, ability to grow in MH agar containing 4 μg/mL ciprofloxacin (clinical resistance breakpoint per CLSI) was used as an indication of FQ-resistance in the present study. This method was used in many of our previous studies and shown to correlate well with the standard MIC-based resistance determination (32, 34, 40, 46, 50, 51). *Campylobacter jejuni* strains (in glycerol stocks saved in freezers) were grown on MH agar at 42°C for 48 h under microaerobic conditions. The ID of all isolates once again confirmed by MALDI-TOF. Then, each culture was transferred to another fresh MH agar and incubated for ~20 h at 42°C. The cells were collected and resuspended in MH broth for inoculation for further *in vitro* analysis.

**Table 1:** Characteristics of *Campylobacter jejuni* strains used in the current study.

Isolate	Source	Origin	Isolation date	Cipro MIC ug/mL^a^	CIP	Reference
ST-93	Feces of healthy cattle	Missouri	2013	0.12	S	(7, 32)
ST-61	Feces of healthy cattle	Iowa	2018	0.12	S	(32)
ST-929 s	Feces of healthy cattle	Iowa	2018	0.12	S	(32)
ST-929r	Feces of healthy cattle	Iowa	2018	4	R	(32)
ST-982	Feces of healthy cattle	Iowa	2018	8	R	(32)
NCTC 11168^b^	Human feces	United Kingdom	1977	0.12	S	(49)

a Ciprofloxacin susceptibility phenotype; R denotes resistant (MIC ≥ 4), S denotes susceptible (MIC ≤ 2).

b Strain used as control.

### 2.3. Growth kinetics of FQ-susceptible and FQ-resistant *Campylobacter jejuni*

A fresh culture of each *C. jejuni* strain was first adjusted to OD_600_ = 0.1 (which corresponds to ^~^10^8^ CFU/mL, as determined previously), diluted 1:100 in MH broth, and 100 μL of the diluted culture was separately inoculated into 3 tubes (with filtered lids to allow air exchange during incubation; Ibis Scientific, NV, United States) with 10 mL of the bovine fecal extract and another set of 3 tubes (the same type as above) with 10 mL of plain MH broth for comparison, yielding an initial bacterial cell density of ~10^4^ CFU/mL (confirmed by viable CFU counts from appropriate serial dilutions inoculated on agar plates for incubation and colony counting). The cultures were incubated together at 39°C under microaerobic conditions to emulate bovine physiological body temperature. To assess differences during the bacterial growth, aliquots of the cultures (100 μL from each of the 3 replicate tubes) were collected at 12, 24, 36, and 48 h of incubation, serially diluted in MH broth as appropriate, and plated onto MH plates for enumeration of bacterial colonies from each replicate tubes separately (3 technical replicates) as described elsewhere (52). Growth curves of the strains were obtained separately in mono-cultures. Two independent experiments (biological replicates) were conducted using the same strains and conditions (6 replicates total per strain per growth medium). No strain genotyping was performed for further confirmatory purposes at this step.

### 2.4. Pairwise competition experiments between FQ-susceptible and -resistant *Campylobacter jejuni* strains

Each of the pairs used in the competition assay contained a FQ-R and a FQ-S *C. jejuni* strain in equal starting concentration. In the first experiment, susceptible and resistant strains were harvested separately in MH broth and adjusted to the same OD_600_ value. Equal volumes of each strain (100 μL) were inoculated together into 3 tubes (the same type as above with filtered lids) with 10 mL of bovine fecal extract and another set of 3 tubes with 10 mL of MH broth for comparison to give an approximate final cell density of 10^7^ CFU/mL for each strain. The cultures were incubated together at 39°C under microaerobic conditions for 24 h and then passaged by transfer of 100 μL of each culture to 10 mL of fresh a medium of corresponding type. To assess the growth differences between the strains, the passages were continued up to 10 times (with 24 h intervals) as described elsewhere (52). Total (susceptible + resistant) *C. jejuni* colonies and FQ-R colonies in each mixture at the end of each passage were determined by serially diluting the mixture in MH broth and transferring 100 μL of the dilution from each tube of the 3 replicate tubes to plain MH plates (antibiotic-free) and ciprofloxacin-containing (4 μL/mL) MH plates, respectively. The number of ciprofloxacin-susceptible cells for each replicate was calculated by subtracting the number of colonies on MH plates with ciprofloxacin from the number of colonies on MH plates without ciprofloxacin. Results (average of 3 replicates) were expressed as the individual growth curves of resistant and susceptible strains. In the second experiment, the initial cell density was reduced to 10^3^ CFU/mL (from 10^7^ CFU/mL) for each strain to evaluate the effect of a lower initial bacterial cell density on the outcome. Two independent experiments (biological replicates) were conducted using the same strains and conditions for each study with different initial cell densities (6 replicates total per strain per initiall cell density per growth medium).

### 2.5. Assessment of FQ resistance development In FQ-susceptible *Campylobacter jejuni* under different cell density and selection pressure

A fresh culture of each of the four FQ-S *C. jejuni* strains (Table 1; ciprofloxacin MIC = 0.12 μg/mL) was separately inoculated into 3 tubes (the same type as above with filtered lids) with 10 mL of bovine fecal extract containing various concentrations of ciprofloxacin (2, 4 or 20 μg/mL) and another set of 3 tubes with 10 mL of MH broth containing the same ciprofloxacin concentrations for comparison. The experiments were conducted with high (107 CFU/mL) and low (105 CFU/mL) initial bacterial cell densities in the culture media. The cultures were incubated at 39°C under microaerobic conditions. Aliquots from each mixture (100–250 μL) were collected at different time points (0, 1, 2, and 3 days of incubation) for CFU counting. Total (susceptible + resistant) *C. jejuni* colonies and FQ-R colonies in each mixture at each time points were determined by using plain MH plates (antibiotic-free) and ciprofloxacin-containing (4 μL/mL) MH plates, respectively. Of note, the detection limit of this method was ^~^4 to 10 CFU/mL. The number of ciprofloxacin-susceptible cells was calculated by subtracting the number of colonies on MH plates with ciprofloxacin from the number of colonies on MH plates without ciprofloxacin. Two independent experiments were conducted using the same strains and conditions for each study with different starting bacterial cell densities and/or ciprofloxacin concentrations (three replicate tubes per experiment).

### 2.6. Statistical analysis

One-way analysis of variance (ANOVA) followed by Tukey’s *post hoc* test was used to calculate the significant differences in growth levels (log-transformed) of each *C. jejuni* strain at each time point (growth kinetics study). Student *t*-test was used to calculate the significant differences in growth levels of FQ-R and FQ-S *C. jejuni* at each time point in the pairwise competition assay, and in the development of FQ resistance mutants from FQ-S *C. jejuni* assay. Differences between the mean values were considered significant at *p* < 0.05. The data was analyzed using GraphPad software (Prism, San Diego, CA, United States).

## 3. Results

### 3.1. FQ-resistant and FQ-susceptible *Campylobacter jejuni* have comparable growth kinetics when individually cultured

**Figure 1:**
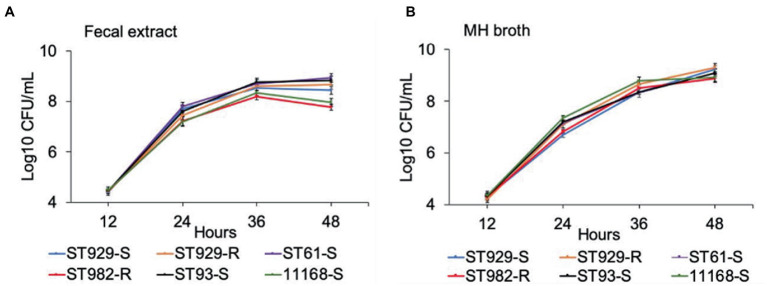
Growth kinetics of FQ-resistant and FQ-susceptible *Campylobacter jejuni* strains grown in bovine fecal extract **(A)** and MH broth **(B)**. FQ-resistant *C. jejuni* ST-982 and ST-929r are represented by the red and orange lines, respectively. FQ-susceptible *C. jejuni* ST-929s, ST-93, ST-61, and NCTC 11168 are represented by the blue, black, purple, and green lines, respectively. The number of the bacterial colonies was measured at 12, 24, 36, and 48 h of incubation. The experiment was repeated twice, and the results of one representative experiment are shown.

FQ-R (e.g., ST-982 and ST-929r) and FQ-S (e.g., ST-929s, ST-93, ST-61, and NCTC 11168) *C. jejuni* strains were separately cultured in antibiotic-free bovine fecal extract (Figure 1A) and plain MH broth (Figure 1B). Although significant differences (value of *p* ≤ 0.05) in growth rates were observed between FQ-R and FQ-S *C. jejuni* strains starting from 24 h of incubation (especially in bovine fecal extract) until the completion of the experiment (Table 2), the strains had comparable growth kinetics overall in both media. There was no distinct growth kinetic pattern in FQ-R strains vs. FQ-S strains in bovine fecal extract, with a mixture of both phenotypes having a relatively faster (ST61-S, ST93-S, ST929-R) or slower (ST929-S, ST982-R) growth. The difference in the growth pattern of FQ-R strains vs. FQ-S strains was even less discernible in MH broth.

**Table 2:** Comparison of the growth kinetics of FQ-resistant vs. FQ-susceptible *Campylobacter jejuni* strains grown individually in bovine fecal extract and MH broth.

	Fecal extract				MH broth			
	12 h^a^	24 h	36 h	48 h	12 h	24 h	36 h	48 h
ST-929 (S)^b^ vs. ST-982 (R)^c^	ns^d^	*p* < 0.001	*p* < 0.001	*p* < 0.001	ns	ns	ns	*p* < 0.001
ST-929 (S) vs. ST-929 (R)	ns	*p* < 0.001	*p* = 0.044	*p* < 0.001	ns	*p* < 0.001	*p* = 0.006	ns
ST-93 (S) vs. ST-982 (R)	ns	*p* < 0.001	*p* < 0.001	*p* < 0.001	ns	*p* < 0.001	ns	*p* = 0.004
ST-61 (S) vs. ST-982 (R)	ns	*p* < 0.001	*p* < 0.001	*p* < 0.001	ns	*p* = 0.002	ns	*p* = 0.002

A *p*-value ≤ 0.05 was considered significant. The blue-highlighted *p-*values represent a faster growth of FQ-S *C. jejuni* strains and the red-highlighted *p-*values represent a faster growth of FQ-resistant *C. jejuni* strains. The data was analyzed using GraphPad software (Prism, San Diego, CA, United States).

a Period of time (hours) after the start of incubation.

b S denotes susceptible (ciprofloxacin MIC ≤ 2).

c R denotes resistant (ciprofloxacin MIC ≥ 4).

d ns denotes non-significant (*p*-value > 0.05).

### 3.2. FQ-resistant and FQ-susceptible *Campylobacter jejuni* strains have comparable fitness

**Figure 2:**
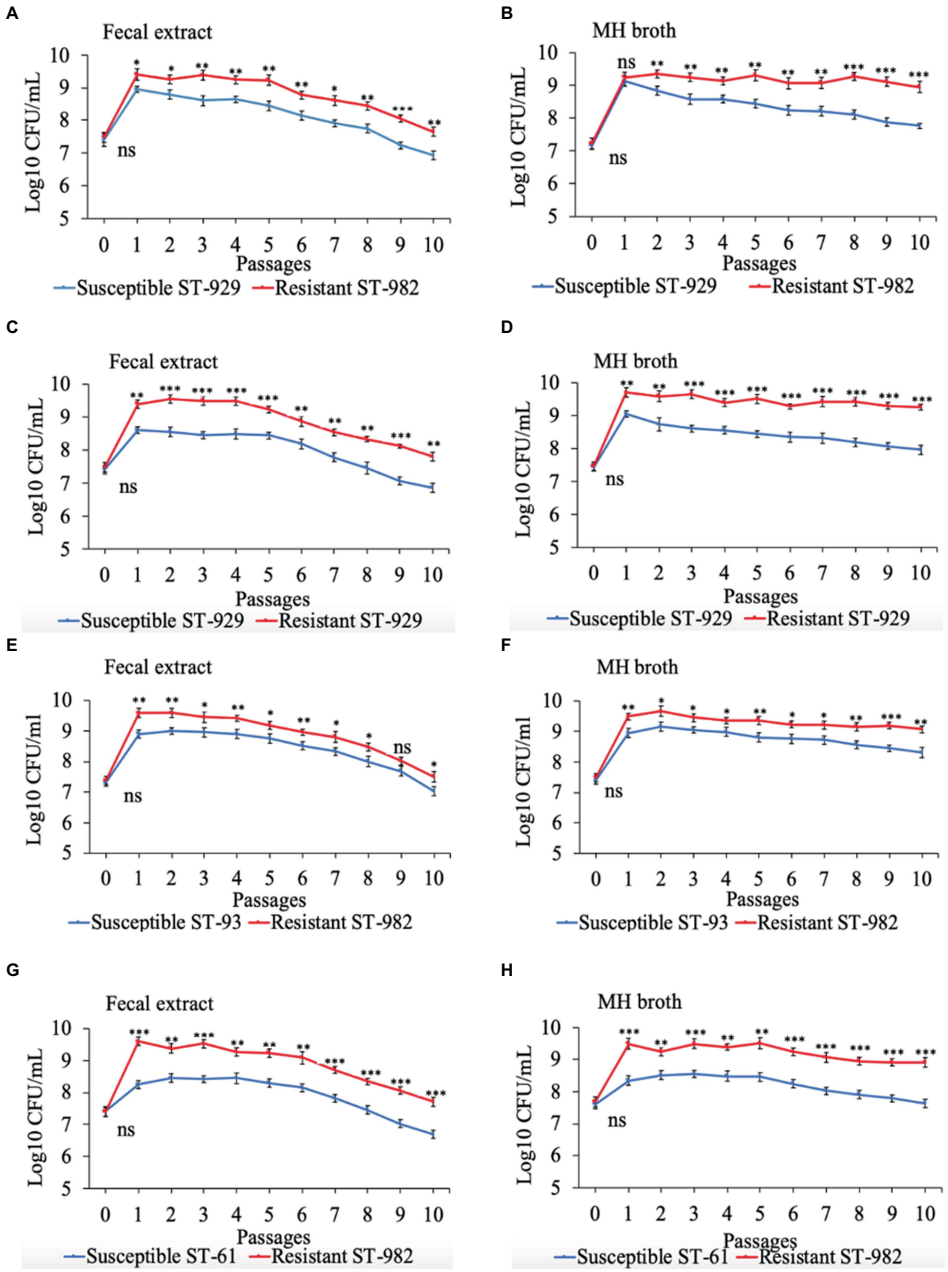
Growth kinetics of FQ-resistant *Campylobacter jejuni* (shown as red lines; resistant ST-982 and resistant ST-929) and FQ-susceptible *C. jejuni* (shown as blue lines; susceptible ST-929, susceptible ST-93, and susceptible ST-61) strains of various genetic background as determined by pairwise competition experiments in mixed culture in bovine fecal extract **(A,C,E,G)** and MH broth **(B,D,F,H)**. The initial bacterial cell density was 10^7^ CFU/mL for each strain. The CFU of each strain at the baseline of each passage was calculated (24 h interval). Significant differences between resistant and susceptible strains are indicated by asterisks: *p*-values less or equal to 0.001 are summarized with three asterisks, *p*-values less or equal to 0.01 are summarized with two asterisks, and *p*-values less or equal to 0.05 are summarized with one asterisk. The experiment was repeated twice, and the results of one representative experiment are shown.

**Figure 3:**
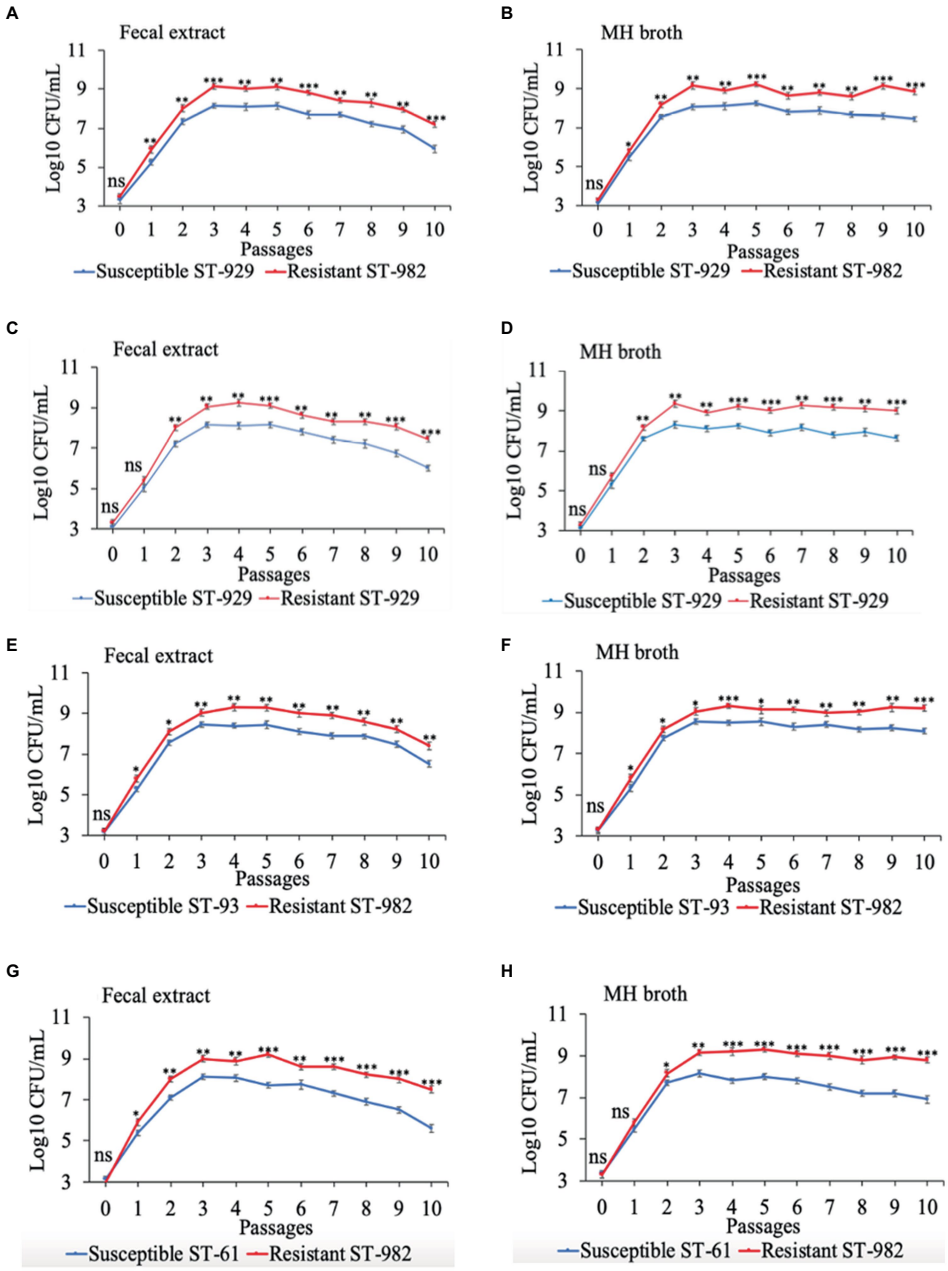
Growth kinetics of FQ-resistant *Campylobacter jejuni* (shown as red lines; resistant ST-982 and resistant ST-929) and FQ-susceptible *C. jejuni* (shown as blue lines; susceptible ST-929, susceptible ST-93, and susceptible ST-61) strains of various genetic background as determined by pairwise competition experiments in mixed culture in bovine fecal extract **(A,C,E,G)** and MH broth **(B,D,F,H)**. The initial bacterial cell density was 10^3^ CFU/mL for each strain. The CFU of each strain at the baseline of each passage was calculated (24 h interval). Significant differences between resistant and susceptible strains are indicated by asterisks: *p*-values less or equal to 0.001 are summarized with three asterisks, *p*-values less or equal to 0.01 are summarized with two asterisks, and *p*-values less or equal to 0.05 are summarized with one asterisk. The experiment was repeated twice, and the results of one representative experiment are shown.

Results of the *in vitro* competition experiments using FQ-R and FQ-S *C. jejuni* strains are shown in Figures 2, 3 as log_10_ CFU/mL for each resistant and susceptible strain during the sequential passages of mixed cultures. Figure 2 shows experiments done using an initial bacterial cell concentration of 10^7^ CFU/mL for each strain, while Figure 3 depicts the experiments done using an initial bacterial cell concentration of 10^3^ CFU/mL for each strain. Interestingly, regardless of the initial bacterial cell concentration employed and different bacterial genotypes used, the growths of the FQ-R *C. jejuni* strains consistently reached higher concentration than those of the FQ-S *C. jejuni* strains throughout the entire experiment, both in bovine fecal extract and MH broth. Although the majority of differences observed were statistically significant, they were relatively of small scale and ranged only between 0.03–1.29 log_10_ CFU/mL in MH broth and 0.07–1.33 log_10_ CFU/mL in bovine fecal extract at high initial bacterial cell concentration (Figure 2), and between 0.015–1.72 log_10_ CFU/mL in MH broth and 0.015–1.9 log_10_ CFU/mL in bovine fecal extract at low initial bacterial cell concentration (Figure 3). Overall, these findings indicated that even though FQ-R *C. jejuni* may have a small fitness advantage over FQ-S *C. jejuni*, a highly comparable growth kinetics was evident between the susceptible and resistant strains during the *in vitro* competition experiments (Figures 2, 3).

### 3.3. Development of FQ resistance in FQ-susceptible *Campylobacter jejuni* strains depends on initial bacterial cell density

**Figure 4:**
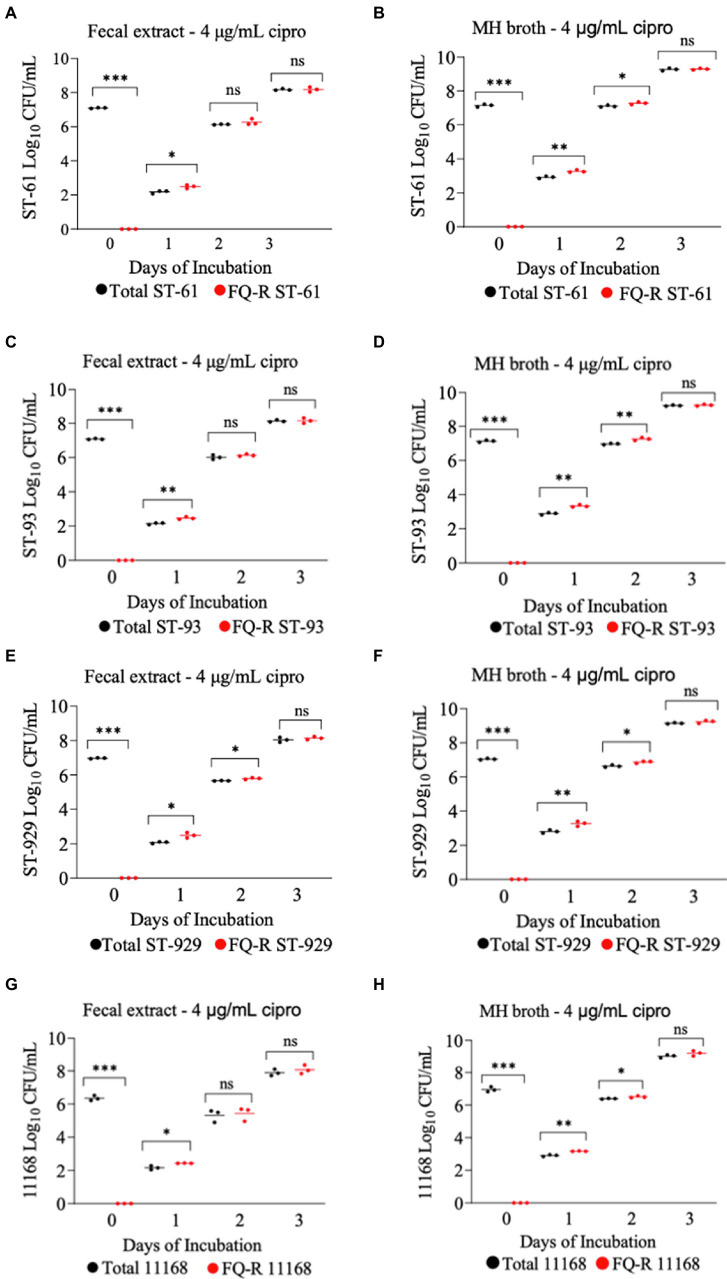
Development of FQ-resistant *Campylobacter jejuni* mutants (shown as red dots) from FQ-susceptible strains (ST-61, ST-93, ST-929, and NCTC 11168) grown in bovine fecal extract **(A,C,E,G)** and MH broth **(B,D,F,H)** supplemented with 4 μg/mL of ciprofloxacin. The initial bacterial cell density (day 0) of each inoculum was 10^7^ CFU/mL. Black dots denote total (susceptible + resistant) colonies. Each dot represents the log_10_ CFU/mL of each strain at a given time point (horizontal bars represent the mean log_10_ CFU/mL of three replicates). The number of bacterial colonies was measured on days 0, 1, 2, and 3 of incubation. The detection limit of the culture was ^~^10 CFU/mL medium. The experiment was repeated twice, and the results of one representative experiment are shown.

All four FQ-susceptible *C. jejuni* strains tested developed resistance to ciprofloxacin within 24 h of incubation in both bovine fecal extract and MH broth (both containing 4 μg/mL ciprofloxacin) when the initial bacterial cell density was relatively high (10^7^ CFU/mL; Figure 4). In big contrast, no FQ-R *C. jejuni* colonies were detected at all throughout the experiment when a lower starting bacterial cell concentration (10^5^ CFU/mL) was used in either growth medium containing the same ciprofloxacin concentration (data not shown). As typically expected, the initial inoculum (10^7^ CFU/mL) of none of the four *C. jejuni* isolates tested had any detectible level of (spontaneous) FQ-R mutants at the start of the experiment (day 0, Figure 4). However, FQ-R colonies appeared as soon as 1 day after the initiation of incubation (day 1) and increased in numbers at the subsequent sampling points (day 2 and day 3, Figure 4). Interestingly, the FQ-R *C. jejuni* population represented virtually 100% of the total colonies detected at all post-incubation sampling points (days 1, 2, and 3) for all 4 strains tested in both bovine fecal extract and MH broth (Figure 4). These results indicated that the initial bacterial cell density significantly and broadly influenced the emergence of FQ-R mutants from FQ-S *C. jejuni* under antibiotic selection pressure (4 μg/mL of ciprofloxacin).

### 3.4. Magnitude of antibiotic selection pressure significantly influences the development of FQ resistance from FQ-susceptible *Campylobacter jejuni*

**Figure 5:**
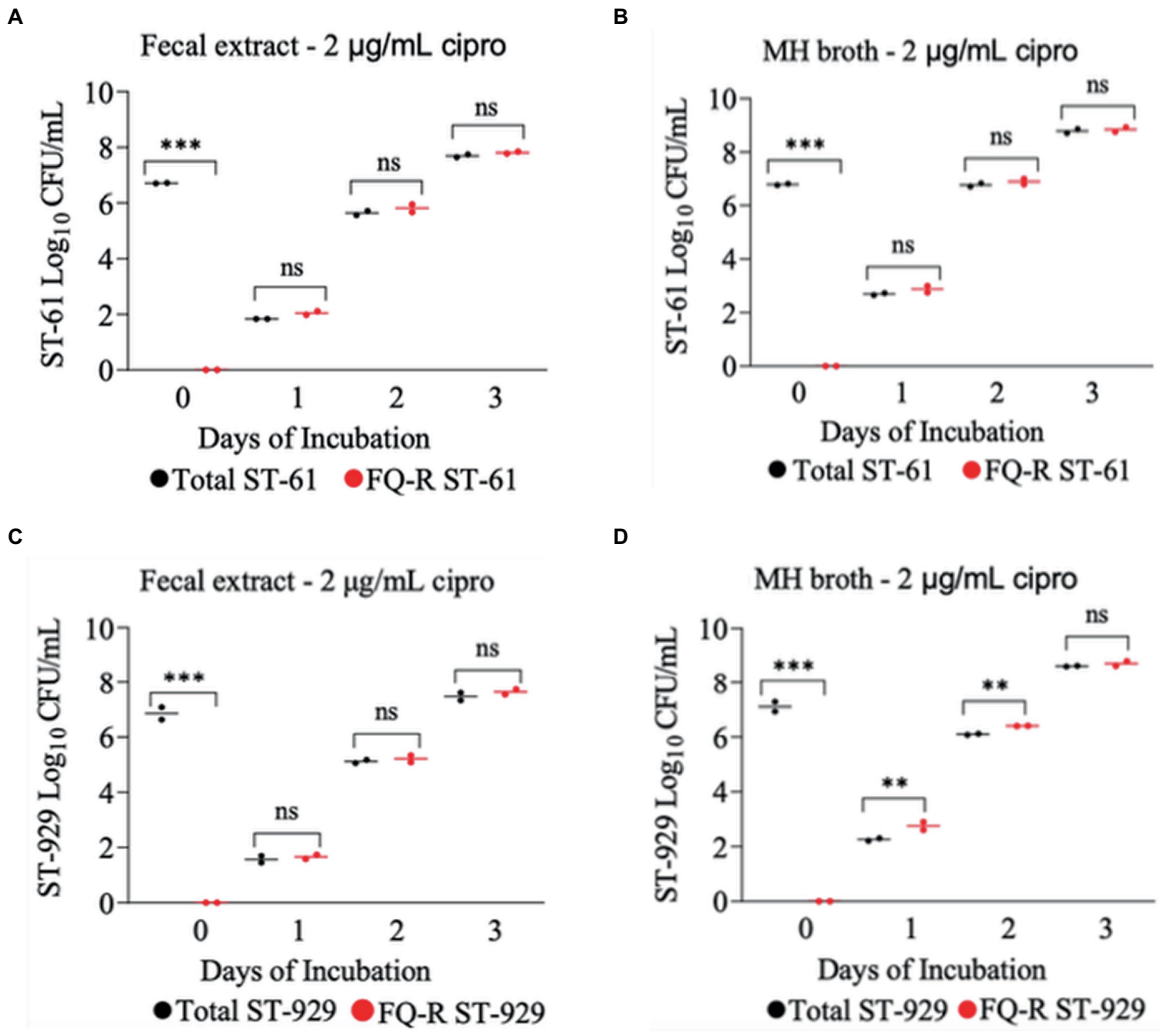
Development of FQ-resistant *C. jejuni* mutants (shown as red dots) from FQ-susceptible strains (ST-61, and ST-929) grown in bovine fecal extract **(A,C)** and MH broth **(B,D)** supplemented with 2 μg/mL of ciprofloxacin. The initial bacterial cell density (day 0) of each inoculum was 10^7^ CFU/mL. Black dots denote total (susceptible + resistant) colonies. Each dot represents the log_10_ CFU/mL of each strain at a given time (horizontal bars represent the mean log_10_ CFU/mL of three replicates). The number of bacterial colonies was measured on days 0, 1, 2, and 3 of incubation. The detection limit of the culture was ^~^4 CFU/mL medium. The experiment was repeated twice, and the results of one representative experiment are shown.

**Figure 6:**
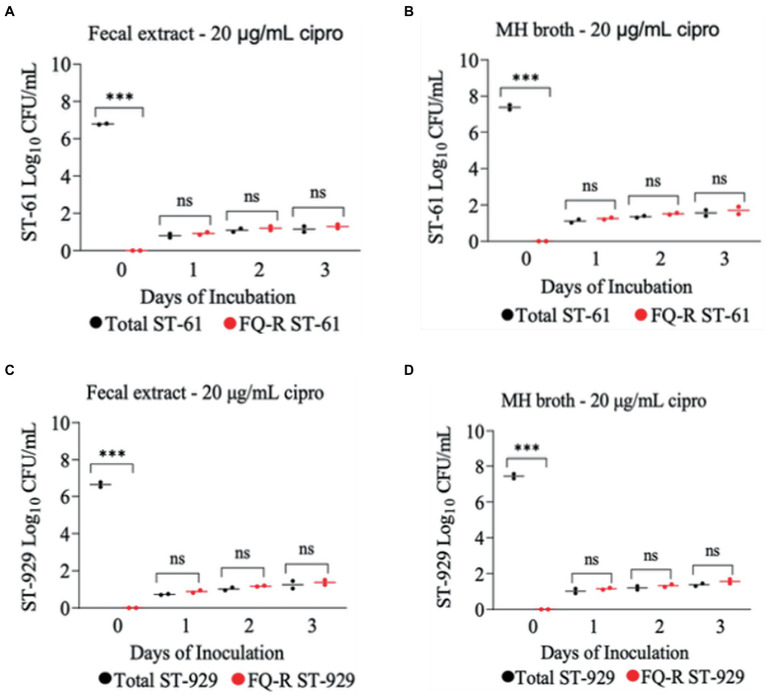
Development of FQ-resistant *C. jejuni* mutants (shown as red dots) from FQ-susceptible strains (ST-61, and ST-929) grown in bovine fecal extract **(A,C)** and MH broth **(B,D)** supplemented with 20 μg/mL of ciprofloxacin. The initial bacterial cell density (day 0) of each inoculum was 10^7^ CFU/mL. Black dots denote total (susceptible + resistant) colonies. Each dot represents the log_10_ CFU/mL of each strain at a given time (horizontal bars represent the mean log_10_ CFU/mL of three replicates). The number of bacterial colonies was measured on days 0, 1, 2, and 3 of incubation. The detection limit of the culture was ^~^4 CFU/mL medium. The experiment was repeated twice, and the results of one representative experiment are shown.

The development of ciprofloxacin resistance in FQ-S *C. jejuni* strains when exposed to 2 μg/mL (Figure 5) followed comparable pattern to that observed when the strains were exposed to 4 μg/mL of the antibiotic (Figure 4). At the beginning of the experiment (day 0) FQ-S strains (10^7^ CFU/mL starting cell density) did not have any detectable FQ-R mutants, as expected (Figure 5). Within a day (day 1) of the exposure to a low dose (2 μg/mL) of ciprofloxacin, FQ-R colonies were emerged from both FQ-S *C. jejuni* strains tested (^~^2 log_10_ CFU/mL) and expanded substantially (^~^6–8 log_10_ CFU/mL) during the course of the experiment (days 2 and 3), with a highly similar pattern in both bovine fecal extract and MH broth (Figure 5). Notably, virtually 100% of the colonies detected were FQ-R at all sampling points after the addition of the antibiotic in the growth medium (days 1, 2, and 3), irrespective of the strain and culture media used (Figure 5). In stark contrast, when FQ-S *C. jejuni* strains (10^7^ CFU/mL starting cell density) were exposed to a higher concentration (20 μg/mL) of ciprofloxacin (Figure 6), only a small fraction (<2 log_10_ CFU/mL) of the original inoculum was able to survive and develop FQ resistance on all of the sampling days (days 1, 2 and 3), regardless of the strains tested and growth medium used. However, similar to what was observed with a lower ciprofloxacin concentration (2 μg/mL; Figure 5), virtually all of the detected colonies were FQ-R (Figure 6).

## 4. Discussion

Over the past decades, *Campylobacter* has developed perpetual resistance to clinically important antibiotics that are used for the treatment of severe cases of human infections, in particular to FQs, posing a threat to treatment efficacy in clinical cases (21, 38, 53). The global predominance of FQ-R *Campylobacter* may have been directly influenced by the frequency with which resistant mutants emerged in response to the selection pressure imposed by the use of antibiotics in both human medicine and veterinary settings (34, 54–59). Notoriously, the transmission and spread of antibiotic-resistant pathogens is not only affected by the emergence of resistant mutants in response to the selection pressure, but also influenced by the relative fitness of the drug-resistant organisms in the absence of selection pressure (39, 40, 60, 61). Cattle are a significant source of human *Campylobacter* infections, and there is a clear trend that FQ-R *Campylobacter* from cattle has become highly prevalent in recent years (7, 15, 62, 63). Even though the selection pressure (use of FQs in cattle) may have contributed to the expansion of FQ-R *Campylobacter*, the actual impact of this factor appears to be rather low (32, 44, 46, 64). In the current study, we examined the hypothesis that the fitness of FQ-R *Campylobacter* may have also played a role in the rise seen in FQ-R *Campylobacter* isolates of cattle origin. By using the FQ-R and FQ-S *C. jejuni* strains collected from calves from our recent study (32), we determined (a) *in vitro* growth kinetics of FQ-R and FQ-S strains in mono-cultures, (b) fitness of FQ-R *C. jejuni* without antibiotic selection pressure, and (c) examined the FQ resistance development in FQ-S *C. jejuni* by using different ciprofloxacin concentrations and initial bacterial cell densities.

Quinolone resistance typically develops at an average rate of 5 × 10^−9^ in *Campylobacter*, with this rate being as high as 5 × 10^−7^ in some strains (65, 66)_._ When *Campylobacter* is exposed to FQs, ciprofloxacin-resistant mutants will likely arise if the cell population is large enough (>10^6^ CFU) (38), suggesting that *Campylobacter* possess a high mutation rate to FQ resistance. Our results are in line with an *in vitro* study conducted previously by our research group (50), in which FQ-resistance emerged readily from FQ-S *C. jejuni* at high (10^7^ and 10^6^ CFU/mL) initial bacterial cell densities when cultured in broth medium containing 4 μg/mL ciprofloxacin though no resistance developed when the initial concentration was10^3^ CFU/mL. Similar findings were also observed in the present study, as FQ-S *C. jejuni* developed resistance to ciprofloxacin (4 μg/mL) within 24 h of *in vitro* exposure at a relatively high initial bacterial cell density (10^7^ CFU/mL; Figure 4), while no colonies of resistant *C. jejuni* strain was detected at a low initial bacterial cell density (10^5^ CFU/mL). In agreement with these *in vitro* data, observations made in animals also indicate the importance of bacterial cell density in the development of FQ resistance in *Campylobacter*. For example, FQ resistance in *Campylobacter* emerges rapidly in chickens but not in cattle under FQ selection pressure, which can, at least in part, be explained by the fact that the organism typically colonizes the chicken intestine at a much higher magnitude (10^8–9^ CFU/g feces) (32, 67) than it does the cattle intestinal tract (10^3–5^ CFU/g feces) (67, 68). In chickens, as soon as 24 h after treatment with FQ antibiotics (enrofloxacin, sarafloxacin, or difloxacin; typically given in drinking water for 5 days), FQ-R *Campylobacter* mutants were found in the feces of treated birds and gradually colonized the intestinal tract at high densities (34, 56, 57). In big contrast, our recent study with calves showed that a single dose s.c. enrofloxacin treatment (7.5 or 12.5 mg/kg) did not result in any detectable level of FQ resistance development from FQ-S *C. jejuni* inhabiting the intestine (^~^10^4–5^ CFU/g feces) of calves (46). Similarly, therapeutic administration of neither oral (20 mg/kg daily for 7 days) nor subcutaneous (20 mg/kg daily for 1–7 days) enrofloxacin resulted in development FQ-resistance in *C. jejuni* NCTC 11168 following experimental inoculation of mice via oral gavage (69).

In the current study, FQ-S *C. jejuni* strains developed resistance to ciprofloxacin more readily when exposed to low levels of ciprofloxacin (2 and 4 μg/mL) compared with exposure to a high level of ciprofloxacin (20 μg/mL). Our data suggest that a high dose of ciprofloxacin is lethal to *Campylobacter*, whereas a low dose may favor the emergence of FQ-R *C. jejuni* from the susceptible strains. Even though it can be rather speculative and cannot be stated with a high degree of certainity, the notion of the mutant selection window (MSW) theory could provide a reasonable explanation for this observation. The range of antimicrobial concentrations known as the MSW ranges from the lowest concentration required to block the growth of wild-type bacteria (MIC) to the highest concentration needed to inhibit the growth of the least susceptible mutant (70). The upper boundary is also known as the mutant prevention concentration (MPC) (71). According to previous publications, the typical MIC of ciprofloxacin in FQ-R *C. jejuni* ranges from 4 to 16 μg/mL (34, 38, 51, 56, 72). Under this theory, the antibiotic becomes lethal to bacteria at concentrations over the MSW, and could no longer select for resistant strains. In the present study, the high level of antibiotic selection pressure might have reached/exceeded the MSW, and thus greatly reducing the emergence of FQ-R mutants in bovine fecal extract and MH broth (Figure 6). In line with this finding, a recent study conducted by our group in which calves were treated with a single dose s.c. enrofloxacin (7.5 and 12.5 mg/kg) found that the drug concentration in the rectal feces of calves had a median of 38–54 μg/g feces for enrofloxacin and 18–21 μg/g feces for ciprofloxacin within 12 h of the injection (73). Notably, in the same study, no FQ-R *C. jejuni* was detected in any of the calves that received enrofloxacin independent of the drug dose used (46). Similarly, we also showed that single dose s.c. danofloxacin treatment in calves colonized with both FQ-R and FQ-S *C. jejuni* resulted in high drug concentration in the rectal feces (median of 382–236 μg/g feces), but did not appear to lead to the development of *de novo* FQ resistance from susceptible strains (32). In contrast to cattle, in a study conducted with broiler chickens, the peak concentration of enrofloxacin was only around 2–4 μg/mL in the intestines of the birds during a standard multi-dose enrofloxacin water treatment, in which FQ-R *C. jejuni* developed soon after the treatment (72). Altogether, these results suggest that the low ciprofloxacin concentrations used in the current study and observed in the intestine of chickens (67, 68) may well have been within the MSW, while the high ciprofloxacin concentrations employed in this study and detected in calf feces (32, 46, 74) may have reached very close to or even exceeded the MPC.

The persistence of antibiotic-resistant *Campylobacter* is influenced by its ability to compete with antibiotic-susceptible strains; this competition dictates whether antibiotic-resistant *Campylobacter* prevails or declines in the absence of antibiotic selection pressure (38). In our study, FQ-R and FQ-S *C. jejuni* had comparable growth rates when individually cultured in either bovine fecal extract or MH broth (Figure 1). Next, we performed pairwise competition experiments to assess the fitness of FQ-R *Campylobacter* by co-culturing several FQ-R *C. jejuni* and FQ-S *C. jejuni* strains of cattle origin in either bovine fecal extract or MH broth containing no antibiotic. Interestingly, FQ-R strains did not have any fitness defect in mixed cultures in the absence of antibiotic selection pressure, but rather displayed a small, albeit significant, growth advantage over the FQ-S strains (Figures 2, 3). Importantly, similar observations were made in calves (from which the *C. jejuni* isolates used here were derived) in our recent study (32), where FQ-R resistant strains were found to coexist with FQ-S strains approximately in equal proportions in the intestinal tract with no antibiotic selection pressure present. Collectively, the findings from both *in vivo* and *in vitro* studies clearly indicate the overall fit nature of FQ-R *C. jejuni* of cattle origin and provide a plausible explanation, at least in part, for the rising trend seen in the prevalence of FQ-R *Campylobacter* in cattle over the past decade.

Our study has some limitations. For example, bovine fecal samples collected from calves in our previous study (46) were stored at −80°C for about 3 years before being used as a bovine fecal extract in the present study. Thus, the storage may have impacted the composition and microbiological properties of the fecal samples. Moreover, the freeze-thawing process (fecal samples were thawed to prepare the fecal extract and then frozen back until further use) may have caused some degree of degradation of the bovine fecal extract. Finaly, even though the bovine fecal extract may be a relevant growth medium to be employed in the experiments performed in the current study, it is important to emphasize that the degree to which it actually mimicked the gastrointestinal tract of cattle is likely to be quite small. Use of digesta instead of fecal extract could have offered more relevant results as it would better mimic the anaerobic conditions in the intestinal lumen. It also should be underlined that caution must be used when extrapolating from *in vitro* results to *in vivo* results and attempting to explain the data with unproven scientific concepts (e.g., the MSW theory).

## 5. Conclusion

Findings from the current study indicate that FQ-R and FQ-S *C. jejuni* strains of cattle origin had comparable growth kinetics and fitness in mono- and co-cultures, respectively. Moreover, FQ-S *C. jejuni* were shown to develop resistance to FQs more readily when exposed to low levels of ciprofloxacin and at a high initial bacterial cell density compared with exposure to a high level of ciprofloxacin and at a low level of initial bacterial cell density. The latter finding suggests that emergence of FQ-R *C. jejuni* mutants from susceptible strains in cattle is likely hampered by both the relatively low level (CFU/g feces) of bacterial colonization and the high level of antibiotic selection pressure in the intestinal tract following the FQ treatment. Altogether, FQ-R *C. jejuni* derived from cattle is found to compete well with FQ-S *C. jejuni* and does not display any fitness defect in the absence of antibiotic selection pressure, providing a plausible explanation for the high prevalence of FQ-R *Campylobacter* in cattle production.

## Data availability statement

The raw data supporting the conclusions of this article will be made available by the authors, without undue reservation.

## Ethics statement

The studies involving animals were reviewed and approved by the Institutional Animal Care and Use Committee (IACUC-18-372) at Iowa State University. Written informed consent from the owners for the participation of their animals in this study was not required in accordance with the national legislation and the institutional requirements.

## Author contributions

DG: methodology, data collection, statistical analysis, preparation of the manuscript. QZ: methodology, funding acquisition, supervision, and preparation of the manuscript. OS: methodology, funding acquisition, supervision, and preparation of the manuscript. All authors contributed to the article and approved the submitted version.

## Funding

This work was supported by the Agriculture and Food Research Initiative’s Competitive Grant 2017-68003-26499 from the USDA National Institute of Food and Agriculture.

## Conflict of interest

The authors declare that the research was conducted in the absence of any commercial or financial relationships that could be construed as a potential conflict of interest.

## Publisher’s note

All claims expressed in this article are solely those of the authors and do not necessarily represent those of their affiliated organizations, or those of the publisher, the editors and the reviewers. Any product that may be evaluated in this article, or claim that may be made by its manufacturer, is not guaranteed or endorsed by the publisher.
